# A ubiquitous method for street scale spatial data collection and analysis in challenging urban environments: mapping health risks using spatial video in Haiti

**DOI:** 10.1186/1476-072X-12-21

**Published:** 2013-04-15

**Authors:** Andrew Curtis, Jason K Blackburn, Jocelyn M Widmer, J Glenn Morris Jr

**Affiliations:** 1 GIS, Health & Hazards Lab, Department of Geography, Kent State University, Kent, OH, 44242, USA; 2 Spatial Epidemiology & Ecology Research Laboratory, Department of Geography, University of Florida, Gainesville, FL, 32610, USA; 3 Emerging Pathogens Institute, University of Florida, Gainesville, FL, 32610, USA; 4 Urban Affairs and Planning, School of Public and International Affairs, Virginia Tech University, Blacksburg, VA, 24061, USA

**Keywords:** Spatial video, Informal settlements, Haiti, Cholera

## Abstract

**Background:**

Fine-scale and longitudinal geospatial analysis of health risks in challenging urban areas is often limited by the lack of other spatial layers even if case data are available. Underlying population counts, residential context, and associated causative factors such as standing water or trash locations are often missing unless collected through logistically difficult, and often expensive, surveys. The lack of spatial context also hinders the interpretation of results and designing intervention strategies structured around analytical insights. This paper offers a ubiquitous spatial data collection approach using a spatial video that can be used to improve analysis and involve participatory collaborations. A case study will be used to illustrate this approach with three health risks mapped at the street scale for a coastal community in Haiti.

**Methods:**

Spatial video was used to collect street and building scale information, including standing water, trash accumulation, presence of dogs, cohort specific population characteristics, and other cultural phenomena. These data were digitized into Google Earth and then coded and analyzed in a GIS using kernel density and spatial filtering approaches. The concentrations of these risks around area schools which are sometimes sources of diarrheal disease infection because of the high concentration of children and variable sanitary practices will show the utility of the method. In addition schools offer potential locations for cholera education interventions.

**Results:**

Previously unavailable fine scale health risk data vary in concentration across the town, with some schools being proximate to greater concentrations of the mapped risks. The spatial video is also used to validate coded data and location specific risks within these “hotspots”.

**Conclusions:**

Spatial video is a tool that can be used in any environment to improve local area health analysis and intervention. The process is rapid and can be repeated in study sites through time to track spatio-temporal dynamics of the communities. Its simplicity should also be used to encourage local participatory collaborations.

## Background

A common geographic constant running though many of the United Nations Millennium Development Goals (MDGs) is addressing health risks associated with informal settlements^a^. The scale of this problem expands when all impoverished urban areas in developing world countries are included because if a settlement has a name, and politically and cartographically “exists”, it can still share many of the telltale signs of poverty associated with informal settlements^b^. These built environment features include temporary or poorly constructed domiciles, narrow alleyways, a lack of basic infrastructure (electricity, paved roads, water and sanitation), access to clean water, and trash accumulations [1]. As a result of these factors, residents of these areas suffer a disproportionate disease burden especially associated with vector and water borne sickness [2-6]. Unfortunately, from a spatial research perspective, it is challenging to acquire the necessary data to perform meaningful intervention-focused analyses at a fine geographic scale. This lack of “official” data may leave researchers with no denominators to calculate rates, independent variables to explain spatial causation, and generally little contextual information to associate with mortality and morbidity [4,7]. Potential solutions include digitizing spatial data from remotely sensed high resolution photography or satellite imagery[8,9], though these sources can be problematic when considering the finest of scales and for densely packed urban environments where extracting individual building characteristics is notoriously difficult [10,11]. Ubiquitous digital earth software, such as Google Earth, has been used to support research in challenging data poor environments, such as designing surveillance systems or spatial sampling frames [12-15]. The creation of spatial data using a volunteered geographic information (VGI) framework, which means the use of individual or organized groups creating spatial data, especially road networks, has also gained considerable attention for filling in spatial data gaps [16,17]. Though even with this latest approach, challenges include maintaining data accuracy and a lack of detail at the finest of scales [18,19]. Traditionally, however, the most common approach to map or gain spatial insight is to conduct field surveys which can be logistically challenging and expensive [1,20,21]. This field-work approach may be linked to grassroot “participatory” mapping projects utilizing the simplest of spatial representations such as sticks and shells, or it can involve teams of researchers using more sophisticated GPS and Smartphone apps [22,23]. This local insight and detail can be invaluable in terms of strategizing interventions, though the cost and logistic framework tend to make these more case specific rather than universal solutions.

This paper presents a methodology to enrich spatial epidemiological analysis as an alternative or complement to the above strategies. An advantage with this approach is that these data retain enough real-world context to allow for survey validation and the reimagining of pre-survey research foci, such as moving from mapping cholera to dengue risk. The simplicity and cost effectiveness of the technique make this method transferable across underserved locations and for multiple time periods, while also facilitating community member participation in the data collection process. As an illustration, this paper will consider three well documented disease-associated health risks (standing water, trash and dogs) and relate them to area schools located in the community. All of these spatial layers, including the school locations, were extracted from the spatial video^c^.

### Spatial video and underserviced urban environments

The challenges of living in underserviced urban environments are well documented: a lack of resources and service provision, little political voice, a high disease burden as a result of poor environmental conditions and high population density, and limited access to health care [24]. Many of these environments are also prone to hazards either due to conditions within (for example the 2013 fires in the informal settlements of Dhaka), proximity to known physical risks (the 2010 earthquake in Haiti) or a combination of both, especially with regard to flooding [25]. It is worth noting that these externalities also highlight another data challenge for studying such settlements; the temporally dynamic nature of construction (and deconstruction), population shifts and cultural activities, which in turn result in spatially variable health challenges, both in terms of analyzing disease or spatially targeting intervention.

This paper is not limited to any one disease investigation, but rather it provides an example of the means to acquire spatial data layers needed for a variety of subsequent analyses. As previously mentioned, arguably the most common approach to collecting data at fine scales is to utilize survey teams. For example, Leptospirosis, caused by *Leptospira interrogans* present in water or soil contaminated with rodent urine, varies temporally with precipitation fluctuations, which could result from seasonality or global weather patterns. To capture this effect, a team went to over 3,600 households to collect a variety of environmental and social data [20]. In their study, three time periods were mapped, with spatial risks being identified as proximity to open refuse and open sewers. Such studies illustrate what can be achieved if large research teams are available. Although these data are invaluable, they still are vulnerable to three main deficiencies. Firstly, it is logistically challenging and expensive. As a universally applied method, this may result in the need to spatially prioritize study areas. In addition, this limits many studies to ask cross-sectional rather than longitudinal research questions with the latter being needed considering the degree of flux in these environments. Secondly, the survey instrumentation limits data collected to the ideas and research questions of that time, with little to no ability to construct different post-survey questions or even validate coding. For example, there may be some crossover between data collected for an informal settlement-focused malaria study if shared with a typhoid research team. However, it is more likely that there will be gaps that still require additional field work. Thirdly, there is usually no visual context to allow researchers to fill-in spaces between the coded locations; data may be collected for two domiciles but the gap between, and the potential linking context is missed. In order to gain more informal settlement-related spatial context Paar and Rekittke [23] employed different technological ground-based approaches to map and visualize improvements, though conceding there was a real need for a Google Street View-type (GSV) method. The spatial video as described in this paper presents such an approach that can be used for both longitudinal and participatory research.

### Spatial Video

The spatial video, which in its simplest form consists of a global position system (GPS)-encoded video can be used to collect high definition video for multiple angles (depending on the number of cameras used), and can be viewed in freeware to display both the image and collection pathway. By developing project specific coding systems, these data can be translated into a geographic information system (GIS) for visualization and analysis, including in combination with other (case or disease related) data layers. Previous spatial video systems utilized by the authors have included proprietary GPS units and specialized software. The GPS receiver would be affixed to the roof of a vehicle that would traverse the study area, with the signal being encoded as an audio stream onto the video. The video would be processed to either run in association with a moving location marker in a stand-alone GIS-lite software, or within Arc GIS 9.3 (for examples of both see [26,27]). Although beneficial for research purposes, this type of system poses challenges in terms of the cost of the cameras, GPS unit and software. These challenges are an impediment for ubiquitous data collection in challenging urban environments. As a result, an extreme sports camera was utilized in this study^d^.

There are four main advantages and one disadvantage in adopting this type of camera as part of a spatial video approach. Firstly, the camera is cheaper than a similar HD camcorder and more lightweight with an inbuilt wide angle high definition lens. This helps data collection in small spaces (such as a narrow roads or alleyways). Secondly, the GPS is built into the camera which reduces the amount of equipment needed such as a roof-mounted aerial, and generally simplifies data collection so that local collaborators can support longitudinal projects^e^. Thirdly, the software available with this camera (Storyteller) is freely available (though a reasonable internet connection is needed to display the Google Maps imagery) allowing for video data to be checked in or close to the field site. This helps identify if any section of the study area has been missed. Another benefit of using freeware is that data can be disseminated and viewed easily without any additional expense. Finally, this particular sports-designed camera is ruggedized and able to handle challenging data collection environments, unlike more traditional camcorders.

A drawback of this system compared to some of the more expensive research-focused kits such as that used by ImageCat (http://www.imagecatinc.com/) is the precision of the GPS. Underserviced urban areas are challenging for GPS signals due to line-of-sight access to satellites in narrow streets with densely packed domiciles. However, even on a wide street, the precision of the GPS may vary in the range of 10 meters. Still, this has not proven too problematic if coding occurs in conjunction with high resolution aerial photography.

### Data Coding

**Figure 1:**
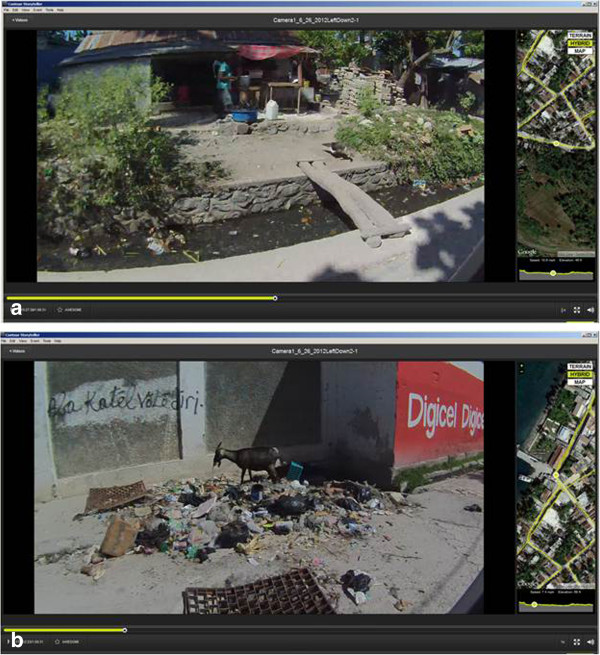
Examples from the spatial video “Storyteller” software showing two of the health risks analyzed in this paper; standing water (a) and trash (b).

Once collected, spatial video can be displayed in the associated software as a main video image, with an accompanying inset map (Figure 1).

In the top image of Figure 1 a typical open water channel or drain is full of water with considerable floating trash. In the background is an open air extension to a habitable structure with cooking taking place in the front yard. Hazards associated with this image could be water borne diseases such as cholera or typhoid, vectored diseases including malaria and dengue, as well as rodent urine in the water linked with Leptospirosis. Previous research into typhoid in Dhaka, upon finding a positive relationship, had suggested the need for replicating similar approaches with smaller water bodies such as puddles because of the roles these often play in domestic chores [28]. *Salmonella typhi* (*S. typhi*) bacteria can remain viable for several days in water so mapping water risk at this scale is imperative to understand ephemeral infection risk. In one video image (not shown here) a child is standing in a similar drain as she collects water from an outflow pipe. In the bottom image a goat feeds on a large trash accumulation. The metal grate at the center of the image suggests trash has washed here during heavy rains. A nightclub is located within fifty feet of this location. In all these images the location of the video frame is shown as a yellow dot on either aerial photography or a more traditional street map. The complete video collection path (a yellow line) and elevation are also displayed.

The next stage is to develop a coding system suitable for GIS analysis. Spatial video coding is a creative process. Some attributes are obvious; for example the presence of water access points, or open roadside sewers. Other attributes are vital but require some form of categorization to assess quantity (amount of trash, depth of flood water or the structural integrity of the domicile). The spatial video can also be used to assess population distribution broken by cohort; the presence of animals, for example dogs (used as a proxy for rats in Leptospirosis research [29]); or characteristics of the built environment (graffiti, or security bars). A further advantage of the spatial video is the ability to return to the source material at a later date to recode new aspects, possibly in association with a different health project.

As the purpose of the methodology presented in this paper is to devise a more ubiquitous data collection approach, Contour GPS video cameras and free display software were utilized, and all digitizing occurred within Google Earth. This was for three reasons. First, Google Earth is freely available. Secondly, Storyteller software uses the same imagery as Google Earth which makes the comparison of spatial features easier for digitizing. Thirdly, the digitization process within Google Earth is more intuitive for non-specialist users as it allows for the mixing of feature types (points, lines and polygons) in a data table-free dialogue box. Features can easily be added, moved, re-edited and deleted. The dialogue box also allows the user to “describe” multiple aspects of the video image. The ease of digitizing within Google Earth has two additional advantages. Firstly, by sending the output KMZ file from Google Earth to the collating researcher, it is possible for non-specialist “field workers” to contribute to what can be called a participatory GIS approach. This has the benefit of including vested parties, while also adding local insight and context. This is not too dissimilar to the participatory frame suggested by [22] or earlier systems developed by this research team [30]. If the coding is done by a researcher, Google Earth still offers advantages as it allows a data strategy to develop through watching the video and not being constrained into populating specific attribute fields until after the coding has been completed. The Google Earth features are imported as points, lines and polygons into ArcGIS 10.1 and converted into shapefiles. Attribute columns are created and populated from the text dialogue accompanying each feature. Notably, in resource limited settings, the ArcGIS steps presented here could be achieved in Quantum GIS, a free open source GIS with much of the same functionality [31].

### Analysis

Once coding is complete, including entry into a GIS, different forms of spatial analysis can be performed. For example, kernel density surface heat maps can be used to develop a general impression of the spatial pattern for any coded variable. Another analytical approach modified from spatial epidemiology is the use of a spatial filter to calculate overlapping rate surfaces where video extracted codes act as both numerators (a health risk) and denominators (all buildings). In previous analyses this approach has been used both as an analysis in itself (a difference of proportions *t* test can be used to assess variations in rates around events being investigated), or the resulting rate layer can be used as input into other spatial models such as Geographically Weighted Regression [32] used to analyze typhoid fever in the Dhaka informal settlements [28].

### Study area

This paper provides an example of one urban environment in Haiti. This urban area, is characterized by shelters made of both solid and temporary walls and roofing systems, open drainage and sewage channels, large trash accumulations, narrow alleyways leading to interior high density housing, spatially variable access to piped water, toilets, and electricity. This coastal town also suffered building collapses during the earthquake of 2010 with a subsequent aftershock being almost directly centered under the town[33]. The lack of earthquake-resilient construction standards coupled with a preponderance of poorly-reinforced concrete resulted in a high number of impact mortalities and morbidities, resulting in lasting effects on housing in the area, as most continue to live in temporary structures provided by NGOs following the earthquake. The town has also suffered hundreds to thousands of cases of cholera since the 2010 epidemic [34], which is unsurprising given the limited access to an improved water source, and the large number of open water drains and trenches[35-38]. Although novel population estimates have been developed for Haiti based on cell phone use [39,40] the post-earthquake population of the town is debatable with estimates ranging from 120,000^f^ to 30,000^g^. Spatial data for the town is also sparse being limited to the road network, a few key buildings (including medical facilities), or other somewhat random places, such as an occasional store location, deemed relevant for a particular project^h^.

The methodology presented here in lieu of a research question is: can the spatial video approach (collection, coding, analysis) provide a more comprehensive fine scale data input layer suitable for inclusion in other modeling frames? More specifically, can it be used to produce a spatial risk assessment for traditional disease related variables around the schools in the community being mapped^i^?

## Results and discussion

**Figure 2:**
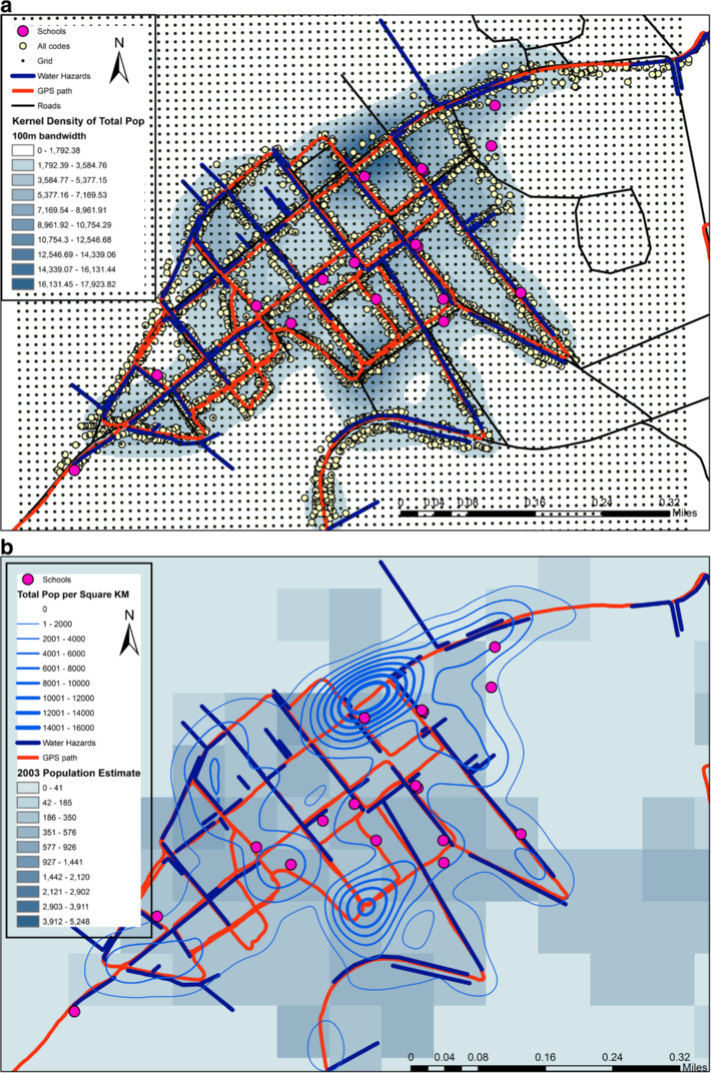
Spatial patterns of coded variables from the video. Kernel density of population, water risks, and school locations (a) and a comparison of spatial video population with alternative pre-earthquake census estimates (b).

Spatial video data collected in June 2012 were digitized into Google Earth. Of four car-mounted cameras, the two angled downwards provided the primary video source because they captured street level water and trash, though for areas with high walls close to the road the horizontal cameras were used to assess building characteristics. A total of 2591 points were digitized in Google Earth, with each location containing multiple attributes (See Table 1). The total number of buildings (ranging from compounds to tents) was 1178. This is approximately 22% of the number of urban houses identified by MINUSTAH ^j^. The variation can be explained by the roads not covered by the spatial video, the amount of building within the interior of city blocks, and structures hidden by roadside barriers such as high walls. A total of 2170 people were digitized along the spatial video path. Only static individuals were coded in order to minimize double counting. This number was further split by age and cohort, with adult males exceeding females, and 53% of the age identified population being children (under 15^k^). Figure 2a displays the GPS path exported from Storyteller, overlaid onto a road layer in ArcGIS 10.1. The yellow dots show locations of all coded points, while the schools have been emphasized in size and colored pink. Linear water hazards (open drainage trenches) are also displayed. The figure also shows the grid output associated with the spatial filter analysis, and a kernel density surface of all people coded. As a comparison, Figure 2b displays the only previously available fine scale (100 meter resolution raster) population estimates taken from the 2003 Haitian census extrapolated using remotely sensed imagery available from the US Census Bureau (http://www.census.gov/population/international/data/mapping/demobase.html). Overlaid on these estimates are the contours of the spatial video coded population kernel density surface.

**Table 1:** A selection of coded data extracted from the spatial video

All digitized points	2591
All Buildings (1 to 4)	1178
Building quality (1 or less)	138
Standing water	827
Water Access	9^q^
All people	2170
Adult males	424
Adult females	340
Children (under 15)	407
Children (under5)	54
Dogs	40
Trash	534
Bars/cafes/restaurants	43
Schools	22

Several decisions on coding were made after the transfer of Google Earth points to ArcGIS. For example, one attribute column included all bars/cafes/restaurants but did not include stores selling food and water, or vendors cooking and selling food on the street (including walking the street or with blankets on the ground). The column for education (and reported in Table 1) included all places of education, ranging from preschool to secondary/high school, and also places of after school learning such as professional schools^l^. However for this paper, only preschools to high schools were analyzed.

**Figure 3:**
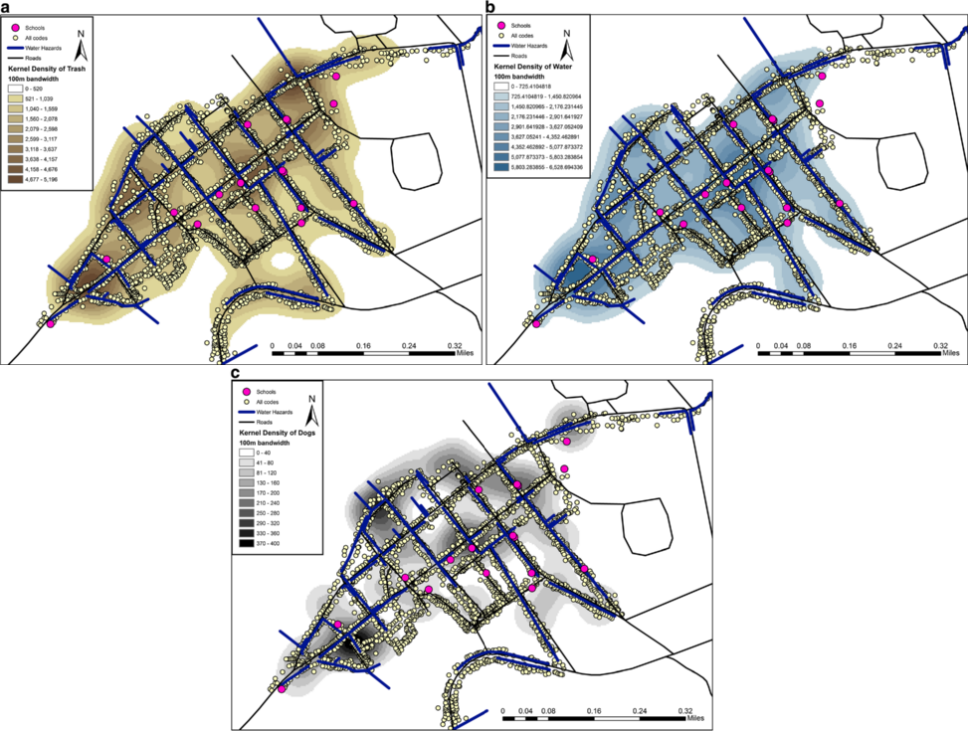
**Kernel density maps of three variables related to health risks extracted from the spatial video.** (**a**) trash; (**b**) standing water; (**c**) dogs.

After coding into ArcGis 10.1, a series of kernel density maps were generated using the quadratic function in the Spatial Analyst Extension. Different bandwidths were chosen, with the results displayed in Figure 3 being 100 m. The justification for this spatial smoothing approach is that coded data does not capture the entire spatial setting of the community, but is rather a sampling of the underlying spatial characteristics; the population distribution rather than a population count^m^. Kernel Density extrapolates based on spatial trends in data rather than simply filling in as would be the case with an interpolation method such as inverse distance weighting.

Figure 3 displays kernel density maps for trash (weighted by amount), flood depth (weighted by depth) and dogs. Five concentrations of trash can be seen in Figure 3a, with two of these also coinciding with the highest weighted standing water concentrations. Interestingly, two of these hotspots also coincide with the highest number of observed dogs. Area 1 and 2 on Figure 3c might be worth further exploration in terms of combined risks, with both containing schools in their highest concentrations.

**Figure 4:**
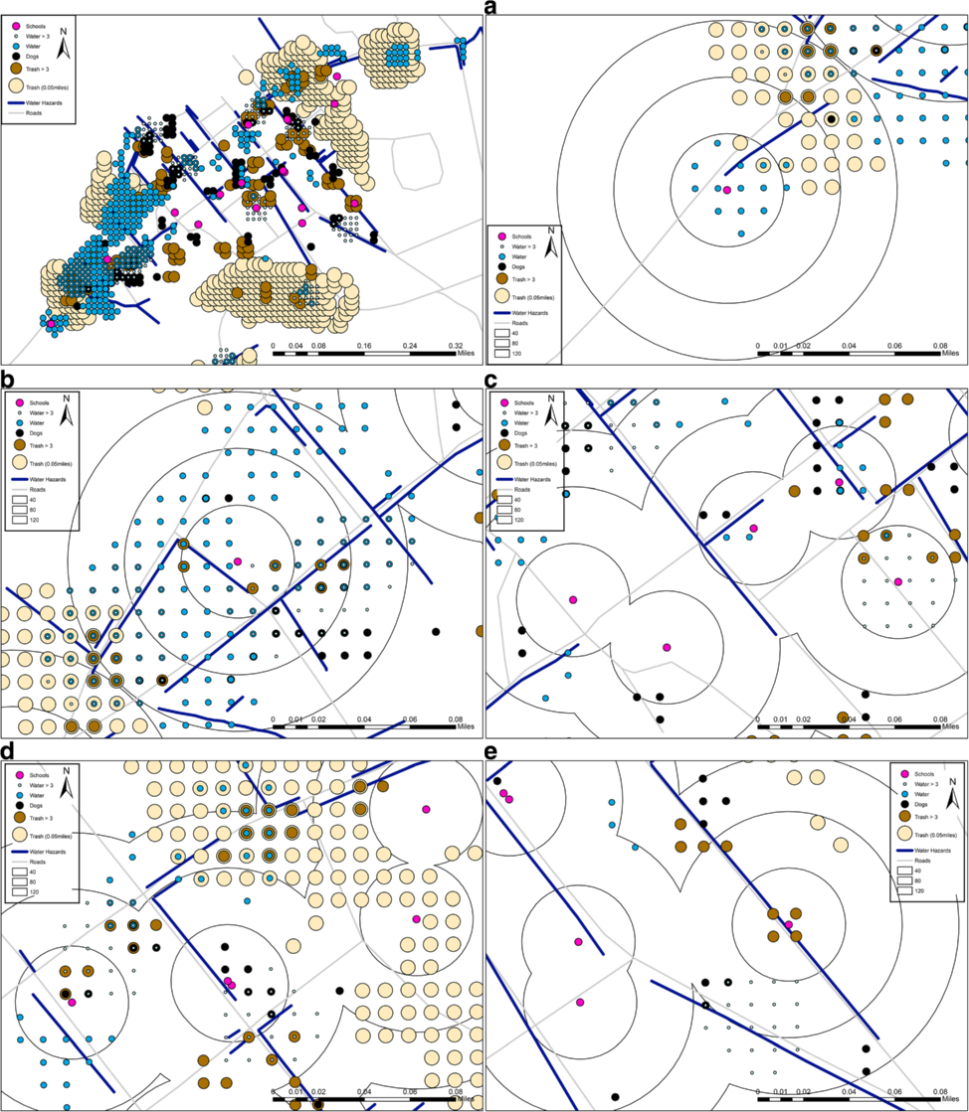
**Health risks coded from the spatial video and mapped as smoothed rates.** Five detail maps (**a** to **e**) show health risk rates (trash, standing water, dogs) as rate nodes within three distance bands (40 80, 120 m) around each school.

The second analysis involved creating a spatial filter grid of the total number of risk locations over a measure of spatial video “effort” (the number of buildings coded). The resulting grid (at 0.16 km as shown in Figure 2a) was joined in ArcGIS 10.1 with different rate output calculations. The nodes displayed in Figure 4 include water weighted by depth (filter size 0.4 km), high water coded above (filter size 0.4 km), dogs (filter size 0.4 km), trash coded above 3 (0.4 km), and all trash (0.8 km). These different permutations are displayed to show spatial consistency across models. In this graphic only the top 50% of all nodes with a rate for that value are displayed (in other words, there was enough of the coded hazard around the node to generate a rate, and the node has a rate higher than 50% of all nodes with such a rate). This is for comparison purposes only and allows the reader to see overlapping concentrations of points as a multiple risk indicator. The area previously identified as “1” in Figure 3 contains risks for all three variables at different filter sizes and data input permutations. In this way the graphic replicates the utility of these grids in the GIS. Indeed, by adding risks together for each node, a measure for total risk can be generated. In this way single or multiple risks can be added to other health investigations, for example, linking the closest node to every cholera location mapped, or to positive pools of mosquitoes, or to serological or water samples taken across the town, or to vaccination locations. Likewise, these methods can be applied to a wider set of zoonoses for the island. For example, Haiti has a well-documented history of human anthrax cases [41,42]. The disease, caused by the zoonotic bacterium *Bacillus anthracis,* is commonly transmitted to humans through contact with infected livestock, particularly goats in Haiti [43]. As illustrated in Figure 1, this methodology can be used to capture and define the human/livestock interface.

Figure 4a to e display the different hazards (high rate nodes and linear water hazards) overlaid onto 40, 80 and 120 m buffers around the schools. These help reveal the fine scale risks surrounding each building or complex. For example, the school in 4A is proximate to both linear water hazards and high risk water nodes within 40 m. Within 120 m several high rate trash nodes are also found. In 4B the school is also proximate to water risks (both linear and high rate nodes). High rate nodes for trash and dogs are also within 120 m. The reader can interpret the remaining maps in a similar way; for example the dominant number of high rate trash nodes around two schools in 4D. What these maps reveal is the specific fine scale risks surrounding each school environment, for example Reis et al. (2008) found a risk of Leptospirosis within 20 m of trash accumulations and open sewers. Not only might these risks threaten the students within (and around) the school, but may also be conduits of infection diffusing outwards. Unfortunately the high concentration of children and variable sanitary practices make schools potential sources of diarrheal disease infection, and proximity to open water should be a concern.

This paper presents a methodology that can be used to build fine-scale spatial data layers for multiple time periods in the most challenging data-poor urban environments. Spatial video offers benefits over more traditional approaches. For example, in this paper as an illustration of how spatial video can be used in an analysis for an underserviced area, three accepted health risks were mapped in association with school locations. A similar analysis might have been performed using data generated from other sources, such as ground surveys. However, the researcher probably could not have returned to the original data to validate key locations, such as the schools. Had each been coded correctly? What type of school (grade level) was it^n^? Were there signs of an active student population? The spatial video allows for such re-interrogating. Indeed, inset images for each school shown on Figures 4a to e were extracted after the analysis. These helped validate buildings that might not be correctly coded or the school is not active, three schools full of students, and others that are for preschoolers. This is a unique advantage of this particular data collection approach, to not only validate key locations but also being able to return to the streets to experience the cultural context or specific risk locations within each hotspot. For example, in the combined multiple risk node hotspot areas identified in this paper, children were playing in and around the drains, people were washing (clothes and pots), and the selling of food was happening either on wooden boards above the drainage trench, or on blankets beside the drain. Activity was also taking place around trash accumulations. Further, it is possible to use this added visual approach as a focal point for brainstorming, either in-person or through multi-site conference using freeware such as Dropbox (http://www.dropbox.com) and Storyteller. Even intervention strategies, such as where to place public information messages^o^ or vaccination strategies can be planned using this approach.

## Conclusion

In summary, this paper has shown the relative ease at which fine scale street-level data can be collected, coded and analyzed in a GIS for challenging urban environments. This opens the possibility for more complex local area spatial analysis and for more longitudinal studies. Repeated transects of communities at fixed temporal intervals can provide insights into the spatio-temporal dynamics of neighborhood infrastructure [44,45]. Apart from collecting otherwise unavailable data, the spatial video also allows researchers to reinvestigate what was collected in the field with more context than previously available. This facilitates brainstorming sessions with academicians who have not visited the site and who may not be aware of local nuances, not just in terms of rephrasing research ideas, but also strategizing interventions such as where to focus education initiatives. This preservation of spatial context also makes these data transferable between research groups who can virtually drive the same streets from a different perspective. It is also possible to imagine the creation of spatial video data warehouses where data are stored, to facilitate research activities at a global level. This would benefit studies focused on the same area, possibly for different time periods, while also allowing cross-site comparisons. These warehouses would also allow future research to return to the archive at a much later date for either comparison purposes or to mine previously un-extracted information from the video.

In order to make comparisons between research areas, data standardization and consistency across non specialist coders is needed [18,19]. Currently efforts are underway by the authors to investigate these issues in terms of using university students to provide a viable means of map making for otherwise data-poor environments. The simplicity of the technology, in combination with ubiquitous geospatial software like Google Earth, also opens the possibilities for on-the-ground collaborators working at distance with academic institutions^p^. This has the quadruple benefit of including vested interests in the project, adding more local insight, initiating research partnerships even without substantial funding. The most labor intensive component of the method described here is the coding, though advances in crowd sourcing strategies may offer opportunities to create near-real time maps.

Another evolution of the approach described here is near-real time access to spatial video by researchers, NGOs or even government departments. The simplicity of the technology and software means that technologically the main impediment to this being the next step is the connection reliability and speed of Internet access. In Haiti a research station associated with the University of Florida provided the necessary Internet link for the data collection described in this paper, permitting immediate data download and quality assessment. The technology also exists for field-based data upload, either using satellite uplink or intelligent sensing of available Wi-Fi. However, the costs of the former and the general lack of Wi-Fi in resource-poor settings such as Haiti limit the utility of these options.

Finally, it is important to discuss the issue of spatial confidentiality and cultural sensitivity. In the United States the type of spatial video recording described in this paper probably would not concern Institutional Review Boards (IRB), especially if these video were not for mass consumption. For other countries and cultures the photography of any facial image is far more sensitive. To address privacy concerns such as these it may be necessary to clearly point cameras to the ground during filming. Arguably the most important data to be captured is at ground-level; trash, standing water etc. Local concerns about privacy may also be alleviated with pre-filming discussions with local officials and community leaders, and, potentially, with use of a vehicle for data collection that is well marked with a statement of purpose. Our data clearly show the potential value of such techniques in development of public health programs and interventions in resource-poor settings; at the same time, it is important that those of us who introduce new geospatial approaches are also the ones that lead this conversation on privacy and ethics.

## Methods

On 26 June 2012 four high definition cameras with internal GPS were mounted on the windows of a field vehicle: two cameras on either side, one with a horizontal field of view, the other pointing downwards to capture curbside data. In approximately two hours most of the Haitian community had been surveyed. Unlike other previous North American projects conducted by the team, hard copy road maps were not available of the town so printed sections from Google Earth were used for navigation along with a hand-held Garmin Oregon device. Only “main” roads on the established grid pattern (though many of these were still unpaved) were chosen, and smaller alleyways avoided. After the day’s data collection, approximately twelve hours of video were downloaded in the University of Florida field station located at the Christianville compound near Gressier. The coordinate path was extracted from the Storyteller software and displayed in Google Earth to check for complete spatial coverage of the town. Three versions of the video data (approximately 3GB data per hour of video) were stored on two ruggedized external hard drives and the original micro SD disk, for transport back to the United States.

The coding process began with an initial viewing of the video to familiarize with the environment before digitizing into Google Earth. Although there was flexibility in what was coded, several elements were captured based on the prior work of the authors, similar literature such as typhoid risk in Dhaka [28], or what was expected for this environment [1,46]. For example, building occupancy and condition ranged from 1 to 4 based on previous recovery metrics [26,45]. This was expanded to also include building robustness which ranged from 0.5 (a temporary structure such as a tent or mix of materials) to 4 (a permanent well-constructed home). Standing water depth and trash (measured on a scale of 1 to 4), along with open sewers or drains were captured because of previously identified associations with disease. For example Dewan et al had found an association between typhoid risk and proximity to water bodies (rivers)[28]. Dogs were also coded because of their previous use as a proxy for rats in Leptospirosis studies [29]. A population count, broken by age (adult, less than 15 years of age, less than 10, less than 5) and sex were recorded partly because of the lack of any post-earthquake population data and because of their analysis in other studies [28]. Other aspects of the built environment were coded either to improve the spatial richness of the social and cultural landscape, to identify potential third spaces (social gathering points which might be useful for vaccination intervention), or locations that could be important in disease spread; these included bars, cafes, restaurants, street vendors, stores, medical facilities and importantly for diarrheal disease spread, schools. At the same time, schools commonly serve as intervention points or to define cohorts for public health studies.

Google Earth offers an easy digitizing option where points, lines or polygons can be drawn on the map and stored in a user specified folder. The user has the option at the end of each session to email a KMZ which can be recombined in a GIS. The dialogue box associated with each point allows for freeform comments to be added without constraint by data fields, though the previously mentioned initial coding strategy was still adopted as a preliminary frame. Points were located on any object of interest; a building, a person (or small group of people), an animal, standing water, water hazard and trash. In addition, lines were added where open drains followed road courses. Additional free form comments were used to contextualize locations or capture additional insights.

One coding decision with a spatial analysis implication is how to code fuzzy space. For example, standing water can be a pool or open drainage trench and the number of points assigned to each, along with the weight (depth), will affect the type of local area analysis used. It was decided to use a two pronged approach. Firstly, water depth was estimated on a four point scale: 1 = any visible water, 2 = substantial water pooling such as centimeters at the bottom of a drain or a road side pool, 3 = an extensive or deep amount of water (the drain was partially full, the puddle’s spatial extent was several meters), and 4 = an unusually deep amount (for example it covered the entire road, or filled a main drainage artery). In Figure 1a the depth of water at this particular location was coded as a “2.5”. Although this is subjective, as with other coding studies of spatial video data, the coder soon develops a high level of consistency across video frames. The final output maps are comparative rather than offering an actual depth measurement – it is possible to visualize the areas of town where trash accumulation was highest. Cross coding validation would be required for multiple coders but for this proof of concept paper only one coder was used. Secondly, the number of points to cover a spatial extent will influence any local area analysis, for example, the centroid of a drainage channel, or three points equally spaced along its length can change hotspot identification. For this purpose, the normal unit of coding was a house, or open space where presumably a house once stood. This was in keeping with other spatial video research [44], and other informal settlement studies which had used domiciles as the unit of analysis (for example the “premise” in Ali et al 2010). The justification for this unit was the potential impact on a single living space. Where no house existed or was visible (for example along a wall), points were placed at approximate house lot separations. The same coding scheme was used for trash, both in terms of the 1 to 4 scale, and the point placement. The trash in Figure 1a was coded as a “2”, and a “3” for Figure 1b.

Once all points and lines had been digitized, the different KMZ files were imported into ArcGIS 10.1. All separate point files were combined, as were the line files. Attribute columns were added to the resulting shapefile and the codes captured in the Google Earth dialogue box split into different fields. Data were visualized using a kernel density analysis, where the input codes (for example water depth) were smoothed using different bandwidths. Kernel density analysis is a commonly applied technique in public health and epidemiology to gauge spatial patterns (usually) of a single variable independent of artificial boundaries. For example the previously described study by Reis et al (2008) applied the technique to subjects with Leptospira anitiobodies in Pau da Lima, Brazil. In their study, bandwidths ranged from 10 to 120 meters. In our study bandwidths of 50 and 100 meters were used though only 100 meters are reported here.

To add more robustness to the analysis, a modification of a technique more commonly used in spatial epidemiology was applied, the DMAP spatial filter [47,48]. DMAP has previously been used by the authors in both health and hazard applications to create smoothed rate surfaces, as an “information layer” where any location can be joined to the closest output node to understand the neighborhood rates (for example the rates of different codes surrounding a school), and as input for different forms of spatial and traditional analysis (see [45]). In this approach the rate of a numerator (the number of dogs) over a denominator (all buildings surveyed which helps standardize the survey “effort”) is generated for user defined overlapping filters (circles). The resulting rate map which is assigned to each grid node can be interpolated to reveal smooth data trends rather than using graduated color mapping within artificial boundaries. Unlike the kernel density maps, these interpolated surfaces display numerator concentrations with respect to the total effort of digitizing along the spatial video routes. In addition, the nodes surrounding key locations can be extracted for analysis, either being compared to each other, or against the whole population. In other projects a test of statistical significance is also added using a Monte Carlo simulation [49]. For this paper each of the schools were spatially joined to the closest rate node, meaning the three risk attributes calculated for that node were assigned to each school.

## Endnotes

^a^See http://www.un.org/millenniumgoals/bkgd.shtml

^b^From this point forward we will call all these urban types “underserved”.

^c^Although other spatial data may be available, it mostly exists in NGO or research silos making its presence and availability largely unknown.

^d^The Contour plus camera and associated “Storyteller” software were used in this paper, see http://www.contour.com

^e^At the time of writing the GIS, Health and Hazards lab at Kent State University has a collaborator collecting data in the informal settlements of Dhaka using a single hand held camera. He collects data within an evolving spatial sampling frame as well as responding to daily events, such as the fires that burnt sections of the informal settlements in early 2013. The flexibility of this camera also allows for hand-held collection which is vital for typical informal settlement environments.

^f^For example, 117504 in the GeoNames database.

^g^MINUSTAH United Nations stabilization mission in Haiti, geospatial data available through http://cegrp.cga.harvard.edu/haiti/?q=resources_data;

^h^For examples see: http://www.gelib.com/haiti-earthquake.htm; http://www.un-spider.org/haiti; http://haitidata.org/data/search.

^i^Schools are used as an example of how these methods can be applied. However we do not intend the reader to make assumptions about the quality of education in this town based on the number mapped. Most have “kindergarten” on them. In general education is as lacking in this area as is WASH infrastructure.

^j^MINUSTAH United Nations stabilization mission in Haiti, geospatial data available through http://cegrp.cga.harvard.edu/haiti/?q=resources_data;

^k^Age was estimated in the coding process so it is possible errors will occur because of malnutrition.

^l^For three locations two buildings were digitized separately though being part of the same large compound – therefore there were 19 unique locations. It might be argued that these additional three buildings should be kept as a potential weight both in terms of the number of children, and in one case, the number of education levels at the same location.

^m^There is a bias to this assumption as only road side structures are recorded and not the interior blocks. Future solutions could include a combination of handheld spatial video paths into the interior, and extrapolation based on coding from high resolution aerial photography.

^n^An interesting aspect with regards schools in Haiti is that students do not necessarily come from the immediate area. This might have implications for the spread of disease because of the mobility tendencies of students to travel some distance. In addition school attendance is quite low compared to the entire population. The National Schools are regarded as being problematic while private schools are quite costly. If the analysis presented here was the beginning of a more in-depth school focused analysis attention would have to be paid to these issues.

**Figure 5:**
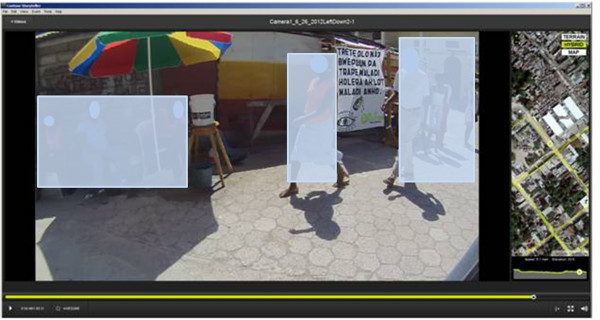
An example of a post-analysis image extracted from the spatial video, in this case public education with regards cholera.

^p^Although this paper uses Haiti as an example, and the hope is that the methods here are translatable across other environments, the authors feel as though the following comment needs to be made. There are certain issues in working in Haiti that have made this long-distance collaboration challenging. Internet connection, access to vehicles, and time needed to be invested in talking through data collection in real time. There is still the need for initial resources and time commitments both on the part of the field workers and on the researcher. Further, each environment will differ with regards to cultural sensitivity in association with this methodology.

^q^There were questionable locations within this category as although a gathering of people centered around bowls or containers no actual water source was visible on the video.

^o^Figure 5 shows one of two public information cholera signs located along the video path, this one in association with an NGO and a vendor with a bucket of water.

## Competing interests

The authors declare that they have no competing interest.

## Authors’ contributions

AC, JB, JW and JGM conceptualized using spatial video to collect fine scale data in Haiti. AC and JW collected data in Haiti. AC performed all coding and analysis. AC, JB and JW wrote the manuscript. All authors read and approved the final manuscript.
